# Modulation of Endothelial Glycocalyx Structure under Inflammatory Conditions

**DOI:** 10.1155/2014/694312

**Published:** 2014-04-03

**Authors:** Hana Kolářová, Barbora Ambrůzová, Lenka Švihálková Šindlerová, Anna Klinke, Lukáš Kubala

**Affiliations:** ^1^Institute of Biophysics, Academy of Sciences of the Czech Republic, Kralovopolska 135, 612 65 Brno, Czech Republic; ^2^International Clinical Research Center, Center of Biomolecular and Cellular Engineering, St. Anne's University Hospital Brno, 656 91 Brno, Czech Republic; ^3^Institute of Experimental Biology, Department of Physiology and Immunology of Animals, Faculty of Science, Masaryk University, Kotlářská 2, 611 37 Brno, Czech Republic; ^4^Heart Center, Department of Cardiology and Cologne Cardiovascular Research Center, University of Cologne, 50937 Cologne, Germany

## Abstract

The glycocalyx of the endothelium is an intravascular compartment that creates a barrier between circulating blood and the vessel wall. The glycocalyx is suggested to play an important role in numerous physiological processes including the regulation of vascular permeability, the prevention of the margination of blood cells to the vessel wall, and the transmission of shear stress. Various theoretical models and experimental approaches provide data about changes to the structure and functions of the glycocalyx under various types of inflammatory conditions. These alterations are suggested to promote inflammatory processes in vessels and contribute to the pathogenesis of number of diseases. In this review we summarize current knowledge about the modulation of the glycocalyx under inflammatory conditions and the consequences for the course of inflammation in vessels. The structure and functions of endothelial glycocalyx are briefly discussed in the context of methodological approaches regarding the determination of endothelial glycocalyx and the uncertainty and challenges involved in glycocalyx structure determination. In addition, the modulation of glycocalyx structure under inflammatory conditions and the possible consequences for pathogenesis of selected diseases and medical conditions (in particular, diabetes, atherosclerosis, ischemia/reperfusion, and sepsis) are summarized. Finally, therapeutic strategies to ameliorate glycocalyx dysfunction suggested by various authors are discussed.

## 1. Introduction

Based on theoretical models and experimental research over the past few decades, it has been shown that the glycocalyx is a multicomponent layer of proteoglycans and glycoproteins covering the luminar endothelium [1–4]. Over the past few decades there has been provided increasing evidence to indicate that the endothelial surface layer plays a considerable physiological role especially in relation to vascular permeability, the adhesion of leucocytes and platelets, the mediation of shear stress, and the modulation of inflammatory processes [2, 5–7]. The vasculoprotective functions of the vessel wall glycocalyx are suggested based on experimental data demonstrating that glycocalyx disruption is accompanied by enhanced sensitivity of the vasculature towards inflammatory and atherogenic stimuli [8, 9]. Detailed descriptions of biological systems and methodologies leading to these conclusions have been published and reviewed previously [2, 3, 7, 10, 11]; therefore, we provide only a brief overview of the glycocalyx functions associated with the modulation of haemostatic and inflammatory processes.

## 2. Structure of Endothelial Glycocalyx

**Figure 1:**
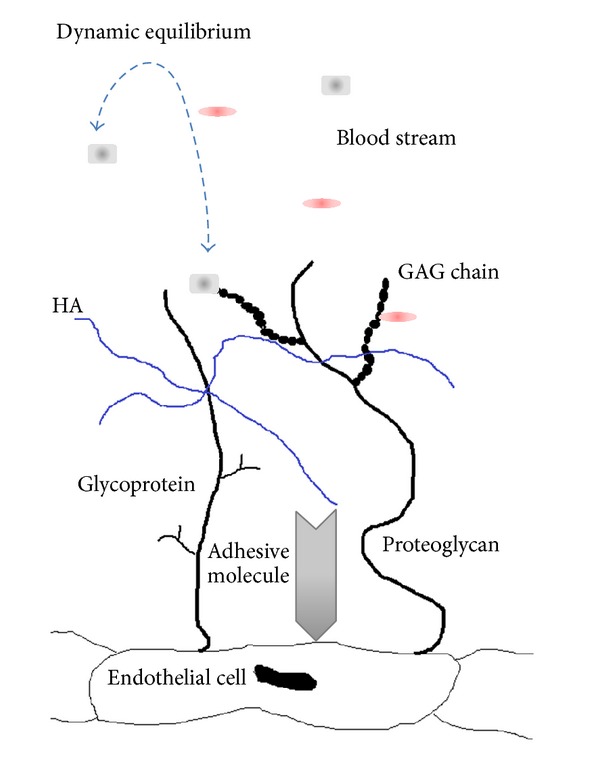
Structure of glycocalyx: the backbone molecules, glycoproteins and proteoglycans; GAG chains linked to core proteins; soluble molecules derived from plasma or endothelium bound to proteoglycans; intertwined HA molecules; sheltered adhesive molecules.

The endothelial cell surface is covered by layer composed of membrane-bound proteoglycans and glycoproteins associated with adsorbed plasma components which are in a dynamic equilibrium with flowing blood depending on the conditions of local microenvironment (Figure 1). Interaction between membrane-bound and soluble components of glycocalyx provides stability of this delicate layer; however, the composition of the glycocalyx is not static; there is a balance between biosynthesis and shedding of glycocalyx components. The backbone molecules of the glycocalyx are proteoglycans and glycoproteins that are mostly bound to endothelial cell surface [2]. On the basis of their structure, they belong to glycoproteins with core proteins of variable size characterized by a long (about 200 sugar residues) unbranched glycosaminoglycan (GAG) side chain linked through* O*-glycosyl linkage. A variety of saccharide motives, including galactosyl residues, as well as* N*-acetyl-glucosamine and* N*-acetylgalactosamine were demonstrated [7].

### 2.1. Proteoglycans

Among proteoglycans present in endothelial glycocalyx are syndecans, glypicans, mimecan (also known as osteoglycin), and biglycan. Syndecans are connected to the cell membrane via a single-span transmembrane domain. Further, they have a short cytoplasmic domain with conserved regions C1 (proximal to membrane) and C2 (distal to membrane) and a variable region V between C1 and C2, unique to the specific syndecan. The size of syndecans varies from 20 to 45 kDa. There are 4 subtypes of syndecans with an extracellular domain specific for each syndecan subtype which binds 3–5 heparan sulfate (HS) or chondroitin sulfate (CS) chains. This domain is variable depending on the structural diversity of the HS and CS chains resulting from a series of posttranslational modifications. The major syndecan in vascular endothelium is syndecan-2. Most of syndecans bind HS; the larger syndecans -1 and -3 can also bind CS. Interestingly, the syndecan ectodomains can be shed from cells and compete for cell surface binding [2, 12–14]. Glypicans have molecular weight around 60–70 kDa. They are connected to the cell membrane via a glycosylphosphatidylinositol (GPI) anchor and, thus, can be released by phospholipase activity [7]. There are 6 glypican subtypes that all share a modular structure with the N-terminal signal sequence, a presumably globular domain containing a characteristic pattern of 14 cysteine residues, a domain with the GAG-attachment sites, and a hydrophobic C-terminal sequence that is involved in the formation of the GPI anchor structure. They bind exclusively HS close to the cell surface [15, 16]. Mimecan and biglycan belong to the small leucine-rich proteoglycan (SLRP) family which binds CS, dermatan sulfate (DS), and keratan sulphate (KS) [17, 18]. The size of SLRP proteins is up to 42 kDa and they are characterized by leucine-rich repeats (LRR) in their central domain. Each LRR has a conserved motif LXXLxLXXNxL, where L is leucine (substitution by isoleucine, valine, or other hydrophobic amino acid is possible) and x could be any amino acid. There are four cysteines with class-conserved domain at the N-terminus of all SLRPs; however, the N-terminus is variably modified in various SLRPs [18]. The SLRP family is divided into 5 classes. Mimecan belongs to class III. Biglycan is a class I member [18]. It is substituted with one or two covalently bound CS/DS chains which are attached to amino acids of the N-terminus [17, 19].

### 2.2. Glycosaminoglycans

GAGs are linear polymers of disaccharides that are composed of D-glucuronic acid, L-iduronic acid, or D-galactose linked to either D-*N*-acetylglucosamine or D-*N*-acetylgalactosamine. GAGs polymers differ in length and are modified by sulfation and/or (de)acetylation to a variable extent. Due to presence of carboxyl and sulfate groups GAGs are of negative charge under physiological conditions [11]. Glycocalyx GAGs except for hyaluronan (hyaluronic acid; HA) are covalently linked to core proteoglycans by coupling of their reducing end with core proteins [2, 20]. In general, GAGs are present in the glycocalyx in the following ratios: HS, more than 50% of the volume; HA forms more than 40%, and DS, around 10%. To a much lower extent, other GAGs such as KS are present in the glycocalyx.

HS is most abundant in the glycocalyx, since HS proteoglycans represent 50–90% of all proteoglycans in endothelial glycocalyx [2]. HS chains can vary with regard to disaccharide composition, domain arrangement, and size. In general, HS can range from 50 to 150 disaccharide units. HS structural diversity is based on a series of enzymatic reactions in the Golgi apparatus, which result in the removal of acetyl groups and sulfation, the epimerization of glucuronic acid to iduronic acid, and the sulfation of the C-6 and C-3 hydroxyl groups of glucosamine and the C-2-hydroxyl groups of uronic residues [21]. The HS chains bind primarily to syndecan ectodomains, often close to the N-terminus. They are connected to the core syndecan protein at a serine-glycine sequence lying within consensus regions rich in acidic residues through a tetrasaccharide linker of xylose-galactose-galactose-uronic acid [12, 22]. Structural diversity defines the functional properties of HS; fine structure appears to be a cell-specific signature, differing between proteoglycans from different sources but not between different proteoglycans from a single source [15].

CS is another abundant GAG in endothelial glycocalyx. The ratio of HS and CS is typically 4 : 1 in vascular endothelium. Type B CS, in which glucuronic acid can epimerize into iduronic acid and influence the functionality, is called dermatan sulfate (DS) and is sometimes classified as a unique class of GAGs [2]. CS is covalently linked to the core protein via the so-called GAG-protein linkage region (GlcA*β*1-3Gal*β*1-3Gal*β*1-4Xyl*β*1-O-Ser) [23]. The number of CS chains linked to the core protein can vary greatly and the MW of CS-GAG can reach up to 3000 kDa [24].

HA is a high-molecular weight not sulphated polymer (MW > 10^6^ Da) that is the only GAG which is not linked to a core protein. It can be bound to the cell membrane and interacts with the cytoskeleton through hyaladherins (e.g., CD44 or RHAMM) [16]. HA is synthetized at the plasma membrane by HA synthases and pushed out of the membrane to the ECM. The configuration of the HA is a pseudorandom coil. HA is highly hygroscopic and, in its aqueous state, is highly viscous and elastic [25].

### 2.3. Glycoproteins

Glycoproteins are another group of “backbone” molecules connecting glycocalyx to the cell membrane. They are glycoconjugates with relatively small (5–12 sugar residues) and branched carbohydrate side chains [2, 7]. Among the most abundant of the functionally important glycocalyx glycoproteins are selectins, integrins, and other adhesion molecules with immunoglobulin structural domains [2, 26–28]. The expression of most of these adhesion molecules on endothelium varies considerably with cell activation or stimulation [2].

Selectins contain a small cytoplasmic tail, a transmembrane domain, several consensus repeats, and an EGF-like domain and a terminal lectin-like domain at the NH_2_-terminus. The last mentioned is responsible for the binding of carbohydrate groups to glycosylated proteins or lipids. The EGF-like domain is involved in selectin-ligand recognition. On the vascular endothelium, E- (64 kDa - calculated from the sequence; different glycosylated forms vary between 100 and 115 kDa) and P- (140 kDa) selectins are expressed which differ in a number of consensus repeats: P-selectin has 9 and E-selectin 6 consensus repeats [2, 27, 28].

Integrins are heterodimeric integral membrane proteins composed of noncovalently bound type I transmembrane glycoprotein *α* (18 known in humans) and *β* subunits (8 known) creating 24 distinct heterodimers. Both of them have large extracellular domains, single-spanning transmembrane domains, and a short cytoplasmic tail [26, 29].

Other glycoproteins harbored within the glycocalyx are adhesion molecules like intercellular adhesion molecules 1 and 2, platelet/endothelial cell adhesion molecule 1, and vascular cell adhesion molecule 1, and glycoproteins acting in coagulation, fibrinolysis, and homeostasis, like Ib-IX-V complex [2].

### 2.4. Soluble Components

A wide range of other molecules are connected to endothelial glycocalyx, such as proteins and soluble proteoglycans that are either derived from the endothelium or from the bloodstream. From a functional view, endothelial glycocalyx harbors proteins involved in inflammation, coagulation, fibrinolysis, and haemostasis mostly connected with the vasculoprotective function of the glycocalyx [25]. They can be physically connected to the glycocalyx by different mechanisms. Firstly, these are receptors or enzymes (e.g., fibroblast growth factor receptor, lipoprotein lipase, and low-density lipoprotein (LDL)).

Another group is composed of plasma-derived molecules binding to the glycocalyx and leading to a concentration gradient (albumin, fibrinogen, orosomucoid). The glycocalyx sieves these soluble components of plasma, establishing a dynamic equilibrium between components in the blood and those retained within the glycocalyx [2, 25]. Finally, there is a group of molecules bound to the glycocalyx structure through interaction with GAGs. In particular, HS proteoglycans contain abundant binding sites for proteins by virtue of specific patterns of sulfation [4]. Among HS chain binding ligands are growth factors (e.g., fibroblast growth factor s, vascular endothelial growth factors, transforming growth factor-*β*, platelet-derived growth factors), extracellular matrix proteins (e.g., fibronectin, vitronectin, collagens, and thrombospondin-1), plasma proteins (e.g., extracellular superoxide dismutase (SOD)), and coagulation inhibitor factors (antithrombin-III, the protein C system, and tissue factor pathway inhibition) [2, 12–14, 30].

## 3. Endothelium Glycocalyx Dimension 

Accurate assessment of the structural organization of normal or damaged endothelial glycocalyx requires reliable visualization techniques [2, 7]. Because of the structural composition of this highly fragile and unstable layer, it is especially challenging to visualize and measure its three-dimensional structure. Primary determination performed using transmission electron microscopy (TEM) suggested a thickness of the glycocalyx layer in the order of tens of nanometers [1, 2, 7, 31]. However, current analysis with the modified fixation of samples for TEM or other methods such as intravital microscopy and confocal microscopy suggested an apparent surface layer thickness of >5 *μ*m [2, 10, 31]. This revealed that initial descriptions had significantly underestimated its actual dimension [5, 6]. Overall, methodological approaches employed to determine the thickness of the glycocalyx have produced widely varying results; thus, this review presents a summary of the various ways in which the endothelial glycocalyx has been visualized up to now.

### 3.1. Electron Microscopy

The first image of the endothelial glycocalyx was obtained by conventional TEM revealing a small layer with a dimension of approximately 20 nm in capillaries [32]. Since then, many subsequent approaches using TEM, along with varying perfusate contents or fixatives, have revealed stained structures on endothelial cell surfaces throughout vessels and cultured cells with large variations in dimension and appearance [8, 9]. In some studies [33, 34], ruthenium red staining in combination with glutaraldehyde/osmium tetroxide fixation was used. However, it is assumed that due to its relatively large molecular size ruthenium red may not gain entry to the entire glycocalyx and may affect glycocalyx geometry by modifying the electrostatic interactions between macromolecules presented on the membrane surface [7]. Interestingly, when specific fixation techniques were applied that stabilize negatively charged structures preventing the loss and/or collapse of these structures, such as lanthanum [34–37] or Alcian blue [35, 38, 39], evidence for a thick endothelial surface layer of up to approximately 800 nm in width was provided [39, 40].

Different results with regard to glycocalyx thickness and structure arising from the use of different TEM approaches may depend on whether the sample is fixed by perfusion or immersion. Chappell et al. documented a dramatic difference between perfusion-fixed and immersion-fixed umbilical vessels, where the latter did not exhibit a glycocalyx [36]. However, the perfusion method with protein free saline can cause shrinkage of the glycocalyx by a partial washout of proteins deposited inside the glycocalyx, which can explain the large discrepancy between* in vivo* measurements and those obtained from perfusion-fixed material [37]. Some studies point to artifacts caused by the presence of aqueous fixatives that may dissolve some structures of the glycocalyx layer. Nevertheless, to obviate this limitation, some groups [41, 42] modified the TEM staining protocol using nonaqueous fixatives based on osmium tetroxide dissolved in a fluorocarbon. This method compensates for techniques requiring washing procedures which can remove plasma proteins attached to glycocalyx structures [2, 7, 43].

However, in all these cases, dehydration by alcohol as a stepping stone towards the embedding of TEM specimens is required. This replacing of the water with organic solvents may lead to a considerable collapse of the hydrated and gel-like state of the glycocalyx [7, 31, 44]. To overcome this problem, Ebong et al., for example, employed the application of rapid freezing and the freeze substitution TEM technique, thus avoiding the use of conventional fixatives. These attempts to preserve the native state of the glycocalyx structure, which has a high water content, resulted in an apparent surface layer thickness of >5 *μ*m [10, 31]. Moreover, this method discerned differences between glycocalyx thicknesses depending on cell type and culture environment [31]. In conclusion, TEM can provide information on the charge, composition, and structure of the glycocalyx; however, results vary greatly according to the fixation and staining methods employed and TEM cannot be used* in vivo* [45].

### 3.2. Intravital Microscopy

Several decades after the first TEM images were made, some studies showed differences between systemic and microvascular haematocrit on cremaster muscle suggesting a glycocalyx layer much thicker than had been indicated using original TEM approaches [1, 8, 9, 40, 46]. The intraluminal distribution of red blood cells (RBCs) in combination with a variety of fluorescently labelled tracer molecules (e.g., dextran) compared with the position of the endothelial wall in capillaries provided a more comprehensive understanding of the exclusion properties of the glycocalyx [2, 47, 48]. The permeation of inert macromolecules into the glycocalyx was determined by both the charge and size of macromolecules [46–49]. Using this methodological approach, the thickness of the glycocalyx in capillaries of hamster cremaster muscle* in vivo* was estimated to be ~0.4-0.5 *μ*m [47].

Techniques measuring the thickness of the glycocalyx on the basis of the depth of infiltration of fluorescently labelled tracer molecules simultaneously enabled the development of other methods such as systemic infusion versus direct perfusion and the use of different species (hamster, mouse, rat) and/or different classes of microvessels (arterioles, capillaries, or venules) [2, 49–51]. One of the main limitations of these methods based on dye-exclusion* in vivo* is the required resolution that is close to the practical resolution limits of* in vivo* optical microscopy. This limits these determinations to microvessels less than 15 *μ*m in diameter [52]. To address this limitation, some authors used high-resolution, near-wall, intravital fluorescent microparticle image velocimetry (*μ*-PIV). The *μ*-PIV technique allows the examination of the velocity profile near the vessel wall in microvessels more than 20 *μ*m in diameter [53, 54].

Finally, the modern clinically applicable microscopic approaches that estimate individual capillary glycocalyx dimensions based on the change in capillary red cell column width following capillary leukocyte passage in microvasculature are orthogonal polarization spectral imaging (OPS, measured in the sublingual area) and its successor, side-stream dark field imaging (SDF, measured on the nail fold) [16, 55–58]. However, these methods also have technical limitations such as the fact that sublingual glycocalyx measurement only gives information on capillary blood vessels [56].

### 3.3. Confocal Microscopy and other Methods

The poor spatial resolution of an intravital optical microscope allows the accurate measurement of glycocalyx thickness only on thin tissues that allow transillumination [55]. Advanced microscopic techniques bring significant improvements which allow the epi-illumination of thicker organ surfaces [2]. Direct visualization of the glycocalyx can be performed via several approaches, mostly using fluorescent labeled lectins that bind specific disaccharide moieties of glycosaminoglycan chains. Fluorescently labeled antibodies recognizing syndecan-1 and fluorescently labeled HA binding protein can be employed in a similar way. Confocal laser scanning microscopy also enables optical sectioning, allowing 3D reconstructions of the specimen. The application of confocal microscopy imaging has revealed a surface layer as thick as 2.5–4.5 *μ*m depending on the size of vessels [2]. However, in general, confocal microscopy is still less suitable for imaging the glycocalyx in arteries due to limited penetration of light with a significant loss of resolution at greater depths (>40 *μ*m) [2]. The arteries must be cut open longitudinally, which might compromise glycocalyx structure [43]. Further, similarly to TEM analysis, the major challenge is the fixation of samples. The preservation of samples using formaldehyde or glutaraldehyde can induce distortion of the glycocalyx because of aldehyde-induced cross-linking of the glycocalyx components and subsequent compression of the glycocalyx toward the cell surface [31]. To eliminate this problem, Barker et al. have applied confocal microscopy to living cells without destroying important functional features of the studied specimens, thereby demonstrating noninvasively the* in vitro* expression of the glycocalyx [59].

A promising technique to directly visualize larger vessels is two-photon laser scanning microscopy (TPLSM), a detailed description of which with respect to its advantages for glycocalyx determination is given elsewhere [2, 43, 60, 61]. Among the main advantages making TPLSM a suitable technique for directly visualizing the delicate endothelial glycocalyx are enhanced penetration depth, good resolution, optical sectioning, and low phototoxicity. When the glycocalyx was imaged with TPLSM in intact mouse carotid arteries, the glycocalyx thickness was found to be 4.5 *μ*m [2].

The mechanical properties of the glycocalyx can be determined using other methods such as atomic force microscopy [62].

### 3.4. Uncertainties and Challenges with respect to Glycocalyx Structure Determination

As described above, there are several major difficulties limiting the demonstration of the three-dimensional structure of endothelial glycocalyx. The main uncertainties arise from comparing the glycocalyx determined using* in vitro* cultured endothelial cells versus* in vivo* vessels and further comparing various methodological approaches.

Concerning cell cultures, overall data suggest that there are more aspects that can modify the formation of the glycocalyx such as the density of cells, culture conditions, cell type, and shear stress [59, 63]. Some studies have demonstrated differences in the structure and composition of the glycocalyx in cells exposed to long-term shear when compared to cells grown under static conditions [10, 62, 64, 65]. Moreover, the comparison of TEM and *μ*-PIV techniques suggests that the thickness of the glycocalyx determined using* in vitro* cell-culture models may be drastically less than the glycocalyx thickness determined* in vivo* [34, 36, 53, 63] despite the fact that cultured cells have been demonstrated to produce some glycocalyx components [7, 66]. Since the validity of using cell culture experiments to study endothelial glycocalyx structure and function has recently been questioned, it remains to be determined whether the structure of the surface layer of cultured endothelium can be used as a model for that seen in* in vivo* conditions [10, 31, 45, 62, 64, 65].

From the viewpoint of methodological approaches, the fixation and/or dehydration of samples during their preparation for TEM analysis is one of the major drawbacks limiting the demonstration of the extent of an* in vitro* endothelial glycocalyx. In addition, the direct visualization of endothelial glycocalyx and the measurement of its dimensions and properties* in vivo* are also a challenge, mainly due to the fact that the endothelial glycocalyx is a very delicate structure depending critically on the presence of flowing plasma.

Interestingly, there are some methods used for the determination of the glycocalyx* in vivo* that are not based on visualization techniques. A generally employed approach to estimate the degradation of glycocalyx* in vivo* is the determination of glycocalyx constituents such as HS, syndecan-1, or HA in plasma [67, 68]. During numerous metabolic (diabetes), vascular (atherosclerosis, hypertension), and surgical (ischemia/reperfusion injury, trauma) disease states, it was shown that the plasma concentration of GAGs increases and that it correlates with the concentration of inflammatory markers [69, 70]. Another interesting method of indirectly comparing the thickness of the glycocalyx is comparing the difference between a noncirculating intravascular volume and a circulating volume using a glycocalyx permeable versus a glycocalyx impermeable tracer [5, 6, 71, 72]. The glycocalyx volume is estimated upon subtraction of these two volumes. Dextran 40 (MW 40 kDa) and indocyanine green are used as the glycocalyx-permeable tracer and a suitable impermeable tracer is a fluorescently labeled erythrocyte. Interestingly, the glycocalyx has been estimated to comprise as much as 25% of the total intravascular space in humans, which would yield an average glycocalyx thickness of up to 2 *μ*m [30, 36, 56, 71–73]. However, the estimated systemic glycocalyx volume does not yield information about the heterogeneity of glycocalyx properties between organs and the technique is still subject to controversy [5, 6].

**Figure 2:**
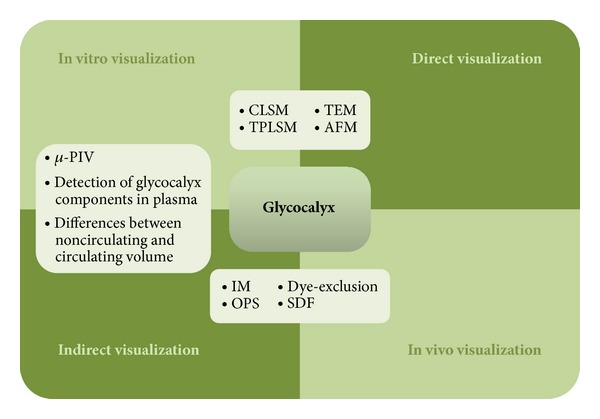
Summary of methods which are currently used in direct or indirect visualization of glycocalyx* in vivo* and* in vitro*. Confocal laser scanning microscopy (CLSM), two-photon laser scanning microscopy (TPLSM), transmission electron microscopy (TEM), atomic force microscopy (AFM), intravital microscopy (IM), microparticle image velocimetry (*μ*-PIV), orthogonal polarization spectral imaging (OPS), and side-stream dark field imaging (SDF).

In conclusion, the heterogeneity of the thicknesses and structures of the glycocalyx reported by various authors arises in part from differences in the applied techniques; the use of* in vivo*,* ex vivo*, and* in vitro* systems/models; differences in sample preparation procedures; the heterogeneity of species and organs; differences in cultivation conditions (Figure 2).

## 4. Glycocalyx Physiological Functions

The glycocalyx layer consists of many highly sulfated GAGs chains providing negative charge for the endothelial surface layer. Due to these electrostatic properties, high molecular density glycocalyx can play a role in the regulation of vascular permeability and fluidic balance through restricting the entry of certain plasma molecules based on their charge, not only on the basis of size and steric hindrance [2, 5, 6, 8–10, 70, 74]. Disruption of glycocalyx can lead to the loss of permeability barrier function with subsequent edema formation. Further, the negative charge can also contribute to repulsion of red blood cells from the endothelium and thus influencing blood cell-vessel wall interaction. Besides this, intact glycocalyx serves as a barrier against the inadvertent adhesion of platelets and leukocytes to the vascular wall [2, 5, 6, 75]. The thickness of the glycocalyx, which is estimated to be around 0.5 *μ*m, exceeds the dimensions of cellular adhesion molecules such as intercellular adhesion molecules 1, vascular cell adhesion molecule 1, and P- and L-selectins and, therefore, attenuating the interaction of blood cells with these molecules [5, 6, 10, 76]. Thus, shedding appears to be required for leukocyte adherence to the vessel wall, because, under normal conditions, leukocytes are supposed to be shielded from contact with their adhesion molecules by the glycocalyx [75, 77].

In recent years, several research studies have put forward the hypothesis that the glycocalyx plays an important role in mechanotransduction [3, 11, 78]. Glycocalyx structures transducing biochemical and mechanical forces into biochemical signals as a consequence of hemodynamic changes are responsible for conformational changes leading to changes in cellular responses. One of these changes is the upregulation of endothelial nitric oxide synthase and the increased production of nitric oxide (NO), which is an important determinant of vascular tone [2, 78, 79]. In any case, the mechanotransduction of glycocalyx is the result of cooperation of all different components.

Further, the importance of the glycocalyx in the protection of endothelial cells against damage by various mediators of oxidative stress was suggested. Under physiological conditions, the glycocalyx contributes to its vasculoprotective effect by docking major enzymatic systems. One of the most important enzymes is extracellular SOD bound to heparan sulphate (HS) proteoglycans, which contributes to a reduction in oxidative stress by quenching oxygen radicals and maintaining NO bioavailability [1, 2, 30].

The glycocalyx is suggested to play an important role in the regulation of coagulation, since a number of mediators involved in the regulation of coagulation pathways, such as antithrombin III, heparin cofactor II, thrombomodulin, and tissue factor pathway inhibitor, are bound within the glycocalyx structure.

Finally, the endothelial glycocalyx can also bind cytokines, which have profound effects on glycocalyx compound synthesis, or modulate inflammatory response by attenuating the binding of cytokines to cell surface receptors [2, 7–9, 30, 79].

All these physiological processes governed by the glycocalyx contribute to the nature of healthy endothelium and vascular homeostasis.

## 5. Inflammation Mediated Alternations of Endothelial Glycocalyx Functions

The vascular endothelium is one of the earliest sites of injury during inflammation. Rapid loss of glycocalyx functions has been directly and indirectly evidenced under various systemic and local inflammatory responses such as diabetes, atherosclerosis, and surgical ischemia/reperfusion injury and sepsis [5, 6, 80]. As discussed above, the functions of glycocalyx are dependent on intact glycocalyx structure. Perturbation of the structure can range from deterioration to fundamental destruction of the glycocalyx layer. The loss of constituents of the endothelial glycocalyx, termed shedding, can encompass selective cleavage of HS and CS or major disturbance represented by removal of entire syndecan and glypican core proteins together with attached glycosaminoglycan side chains [5, 6]. The shedding of the glycocalyx in response to inflammatory mediators such as cytokines and chemoattractants was found to occur in arterioles, capillaries, and venules under various experimental models of inflammation [5, 6, 80]. Although at first sight, a reduction of the glycocalyx might appear favorable for nutrient supply, the microvascular changes associated with loss of the glycocalyx and impaired vascular protection could be negatively affect vessel functions [4]. For amplification of inflammation seems to be crucial the release of inflammatory mediators which could initiate the accessibility of leukocytes to adhesion molecules by degrading the enveloping glycocalyx, as was reviewed in Chappell et al. [80]. Based on mechanism of action, inflammatory mediators can directly affect endothelial cells that in response alter glycocalyx structures. Additionally, under inflammatory conditions, activated subsets of leukocytes such as polymorphonuclear leukocytes, macrophages, and mast cells degranulate enzymes which then can contribute to degradation of the glycocalyx [4, 81].

### 5.1. Mediators Released during Inflammation Contributing to Glycocalyx Destruction

Upon their activation, inflammatory cells release a wide range of enzymes and reactive species which can contribute to glycocalyx damage. In particular, activated neutrophil granulocytes, the most abundant blood leukocytes in humans, can induce glycocalyx damage by producing reactive oxygen and nitrogen species (ROS/RNSs) and releasing proteases from their storage granules [70]. Moreover, mast cells, a less abundant leukocyte subset, can release heparanase directly with a significant potential to disturb glycocalyx structure through the degradation of HS [5, 6, 79, 80, 82–84].

It is recognized that ROS/RNSs are capable of degrading HA, HS, and CS. Not all ROS/RNSs are a direct threat to the glycocalyx. The fragmentation of the glycocalyx is mediated by tertiary species, such as the hydroxyl radical or hypohalous acids, which are formed by the catalysis of neutrophil-derived MPO bound to the negatively charged GAG chains [70]. Further, it is supposed that ROS/RNSs are capable of attacking the glycocalyx generated in the direct vicinity of the endothelial lining [70]. The cleavage of HS after ROS/RNSs coupled with an increase in macromolecular passage follows a similar pattern seen after treatment with HS degrading enzymes [38, 85]. Moreover, the protein core of proteoglycans can also be subject to oxidation/nitrosation and these oxidative and nitrosative modifications at the level of proteoglycans could negatively affect glycocalyx integrity [70]. Further, ROS/RNSs not only pose a direct threat to the glycocalyx but additionally could potentiate the proteolysis of the glycocalyx via the activation of matrix metalloproteinases (MMPs) and inactivation of endogenous protease inhibitors [70].

Proteases are important mediators released and activated during inflammatory conditions that pose a significant threat to the glycocalyx [76, 86]. These include especially MMPs and neutrophil elastase. MMPs are stored within vesicles of phagocytes and the endothelium and after appropriate stimulation are released and activated [87]. The inhibition of MMP activity by doxycycline significantly reduced shedding of the glycocalyx [86–88]. It was suggested that cleavage of the entire syndecan ectodomain with the attached GAG branches by MMPs may be responsible for shedding of the glycocalyx [86, 87]. Interestingly, MMPs were shown to have a high affinity to HSs and this HS-mediated immobilization of MMPs on glycocalyx components underlines the destructive potential of MMPs [89]. Moreover, MMPs were suggested to directly cleave GAGs, HS proteoglycan syndecan-1, and the HA receptor CD44 [90–92].

Besides MMPs, the importance of neutrophil elastase in the degradation of endothelial glycocalyx under systemic or local inflammation conditions is suggested. Neutrophil elastase is released from the azurophilic granules of neutrophil granulocytes after activation. Neutrophil elastase can bind to the HS branches of syndecan probably via interaction with the sulfate moieties and is capable of syndecan degradation [70].

Overall, as described above, inflammatory mediator-mediated alterations of the glycocalyx structure or its shedding are best described as a result of the release of proteases and other degrading enzymes from different types of phagocytes and endothelial cells themselves [93].

### 5.2. Other Mediators

Further, glycocalyx degradation could be induced by low and/or turbulent shear stress or exposure of the endothelium to oxidized low density lipoprotein [94, 95]. It was also suggested that endothelial cells directly respond to inflammatory mediators such as tumor necrosis factor *α* (TNF-*α*) or bacterial lipopolysaccharide by shedding the glycocalyx [36, 67, 81, 93, 96]. This is possibly due to the activation or release of intracellular or membrane-bound degradation enzymes such as proteases [11, 76, 80].

## 6. Pathophysiological Implications of Glycocalyx Alternated Structure and Functions 

**Figure 3:**
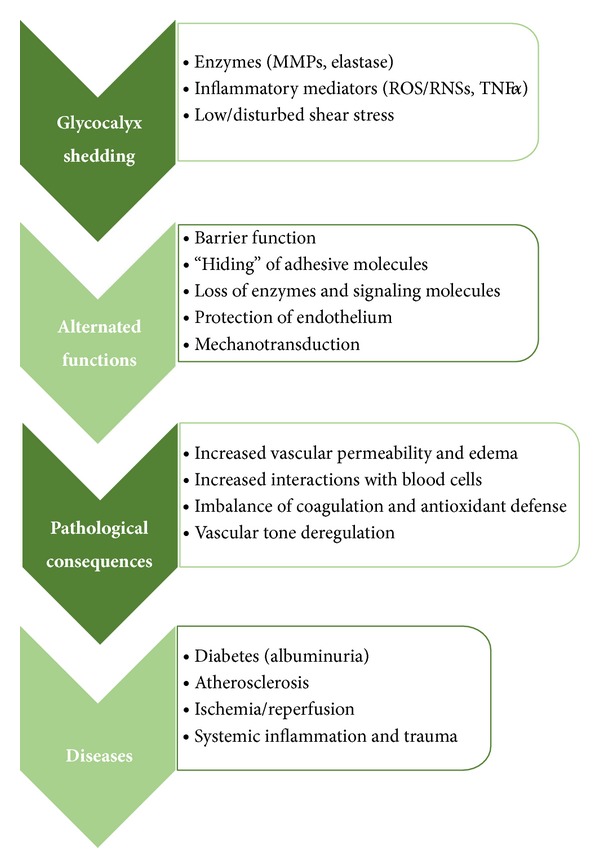
Overview of alternated glycocalyx functions as a result of its shedding which could lead to pathological states connected with various diseases. Matrix metalloproteinases (MMPs), reactive oxygen and nitrogen species (ROSs/RNSs), and tumor necrosis factor *α* (TNF-*α*).

As was summarized above, under physiological conditions the glycocalyx contributes to the regulation of vascular permeability and tone and serves as a barrier deterring leukocyte adhesion [70]. Thus alterations of these functions are associated with a wide range of pathophysiological consequences such as capillary leak syndrome, edema formation, accelerated inflammation, platelet hyperaggregation, hypercoagulation, and loss of vascular responsiveness [11]. In general, it is believed that vascular sites with diminished glycocalyx are more vulnerable to proinflammatory and atherosclerotic consequences [1, 4–6] (Figure 3).

The importance of the loss of glycocalyx barrier functions leading to increased vascular permeability was suggested from experiments showing that glycocalyx degradation is associated with a reduction in the exclusion of anionic dextrans [49], with an increased protein permeability [97], with an increased glomerular clearance of albumin [98, 99], and with the formation of perivascular edema [39]. Further, it is suggested that the loss of the glycocalyx uncovers membrane surface adhesion molecules that potentiate leukocyte adhesion to the vessel wall [5, 6]. Thus, the increased adhesion of leukocytes and increased vascular permeability are the main experimentally confirmed consequences of damaged glycocalyx barrier function. Interestingly other mechanisms underlying this phenomenon of increased vascular permeability were suggested, including a loss of barrier function and induction of the rearrangement of intercellular endothelial junctions. In this case, signalling through syndecans integrates extracellular signals by their association with cytosolic effectors, leading to the rearrangement of cytoskeletal proteins and altering intercellular junctions, which become more permeable, allowing fluid extravasation [69].

Further, some of the pathological consequences are supposed to be due to the loss of different enzymes and signaling molecules stored in the glycocalyx structure, including SOD, antithrombin III, and thrombomodulin [8, 9, 79]. This perhaps contributes to an imbalance in enzymatic systems such as coagulation and antioxidant defense [79]. Finally, the pathological consequences of a loss of mechanotransduction functions due to damage of the glycocalyx structure are discussed by various authors [1, 3, 11, 79, 100, 101].

Focusing on inflammatory reaction, it should also be appreciated that degradation of the glycocalyx by inflammatory mediators and the release of its fragments into the circulation can significantly contribute to the potentiation of inflammatory processes, starting and maintaining a potentially destructive feed-back mechanism. The shed HS and HA fragments, which may be released with glycocalyx disruption, are suggested to act as pro-inflammatory molecules with, for example, significant chemotactical properties [16, 69, 102, 103].

## 7. Pathological Conditions Related to Altered Glycocalyx Structure

The alterations of functions of the endothelial glycocalyx layer are involved in many inflammation-based pathological states. Recent studies in humans revealed the degree of glycocalyx shedding to depend on the extent of the inflammatory state and that there are correlations between the severity of a disease and the level of glycocalyx components in blood [69, 70, 104]. The importance of glycocalyx changes in chronic inflammatory reactions was highlighted, suggesting that the vessel wall in patients with chronic inflammatory diseases is more sensitive to, for example, proatherogenic stimuli [79]. In this review we will discuss more deeply conditions such as diabetes, atherosclerosis, ischemia/reperfusion, and sepsis, in which inflammatory disorders take place.

### 7.1. Diabetes

Diabetes mellitus is a clinically well-defined metabolic disease connected with insulin absence or resistance and subsequent hyperglycemia. Patients with diabetes mellitus revealed a tendency to develop vascular complications, such as microalbuminuria, retino- and nephropathy, and elevated risks of atherothrombotic cardiovascular events [2, 74]. Fundamental pathogenic mechanisms in diabetes-associated vascular disease include accentuated vascular inflammation and increased oxidative stress [105]. These complications are suggested to be related to altered vascular functions such as enhanced endothelial permeability and impaired NO synthase function, indicating the compromised protective capacity of the vessel wall [71, 72, 74, 106–109]. Interestingly, it can be suggested that these typical pathological manifestations of impaired endothelium function in diabetes mellitus patients exhibit signs of glycocalyx degradation [71, 72, 110]. In the glomerulus, damage to the endothelial glycocalyx can alter the permeability of capillary beds, which is clinically apparent as albuminuria accompanied by increased systemic microvascular permeability [106]. It was shown that the changes in glomerular endothelial glycocalyx in early diabetic nephropathy are consistent with the early loss of charge selectivity that occurs in animal models of diabetic nephropathy and in individuals with type 1 and type 2 diabetes and microalbuminuria [98, 99, 111]. Hyperglycemia-induced glycocalyx degradation has been repeatedly demonstrated by a number of authors, who provided data that acute and long-term hyperglycemia is associated with the profound thinning of the glycocalyx and glycocalyx degradation as determined by various methods [71, 72, 112].

Several molecular mechanisms are suggested to be responsible for the reduction of glycocalyx structure in diabetic patients. Primarily, some authors speculate that it is directly caused by increased levels of glucose, since many of these effects can be experimentally induced in nondiabetic subjects as well. Interestingly, even short-term vessel perfusions with increased glucose above physiological levels were associated with vascular function alterations such as shear stress-induced arterial dilatation and vascular permeability, which could be connected with glycocalyx degradation [72, 74, 107–109]. It was suggested that glucose can influence glycocalyx structure because of the glycoproteinaceous nature of the glycocalyx with which glucose interacts [72]. Another suggested mechanism is connected with altered HS biosynthesis, since this was severely disrupted by the exposure of human glomerular endothelial cell monolayers to high glucose concentrations, these being associated with disrupted endothelial glycocalyx structure and the increased passage of albumin across the monolayer [113]. Finally, the relationship between HA metabolism alternations and hyperglycemia related vascular dysfunction was recently reviewed by Lennon and Singleton [25]. Plasma levels of HA and hyaluronidase were found to be elevated in patients with diabetes, reflecting the increased synthesis and shedding of HA under hyperglycemic conditions [71, 72, 112, 114].

Further, not directly related to glycocalyx structure alternations, inflammatory mediators can also increase endothelial cell-cell junction width and in some cases induce parajunctional transcellular holes, associated with increased permeability as was shown in both animal models and patients with diabetes [115]. Further, hyperglycemia-mediated damage to the podocytes may result in reduced vascular endothelial growth factor production, which leads to glomerular endothelial cell dysfunction and potentially also to glycocalyx disruption [116].

### 7.2. Atherosclerosis

Atherosclerosis is a large artery disease, of which the initiating step in pathogenesis is vascular endothelial barrier dysfunction. This is followed by the subendothelial retention of atherogenic lipoproteins, cholesterol, and monocytes forming a plaque that is marked by disturbed flow profiles. Subsequently, there is augmented endothelial barrier dysfunction and vascular smooth muscle cell proliferation, eventually leading to plaque rupture and thrombosis [25, 48, 95, 117, 118].

In general, the role of the endothelial glycocalyx in atherogenesis is, as yet, not well established, but there are some interesting observations which point at its involvement. The glycocalyx was visualized in large arteries in different animal models suggesting that the glycocalyx could add to the vasculoprotective properties of the vessel wall in the macrovasculature [30, 119]. The protective functions of the glycocalyx are suggested from research experimentally decreasing glycocalyx formation through the inhibition of HA synthesis, which led to the increased adhesion of leukocytes in the carotid artery of ApoE deficient mice and ultimately to increased atherosclerosis [117]. Interestingly, the thickness of the glycocalyx decreases in high-risk regions of the murine carotid artery (sites within the arterial system which have low or disturbed shear rates) compared with low-risk regions, supporting the potential role of glycocalyx disruption in rendering disturbed flow regions more susceptible to atherogenesis [30, 120]. Besides shear stress, another risk factor which has been shown to impair vascular glycocalyx is the ratio of oxidized lipoproteins to LDL [95]. Vink, van den Berg, and colleagues showed a disruption of the glycocalyx induced by a high-fat and high-cholesterol diet or by an administration of clinically relevant doses of ox-LDL evoking, for example, increased platelet adhesion in hamster cremaster muscle microcirculation [95, 118]. In addition, exposure of endothelial cells to oxidized LDL* in vitro* decreases the amount of HS proteoglycans associated with the luminal cell surface. The loss of glycocalyx resulted also in the shedding of endogenous protective enzymes, such as extracellular SOD, and increased the oxidative stress on endothelial cells. Interestingly, the importance of ROSs in this process was underlined by the fact that SOD and catalases coinfusion annulled the effect of ox-LDL [95].

Overall, it can be suggested that the glycocalyx layer plays an important role in the protection of large vessels against atherothrombotic disease and that alterations to endothelial glycocalyx are involved in the initiation and progression of the atherosclerotic process.

### 7.3. Ischemia/Reperfusion

Ischemia/reperfusion injury leads to tissue damage caused by blood flow restoration to a tissue/organ after a period of disrupted blood flow inducing total or partial ischemia. Although the extent of the damage resulting from ischemia/reperfusion varies between tissues, a common component of this pathological process is microvascular dysfunction [2, 7, 93, 121]. In particular, in postcapillary venules, endothelial cells suffer from increased oxidative stress, leukocyte adherence and transmigration, and vascular permeability [2, 122]. The involvement of alterations to the endothelial glycocalyx in this process is supported by data showing that ischemia/reperfusion injury can stimulate shedding of the glycocalyx [7, 93, 121–123]. As was shown by Mulivor and Lipowsky, intestinal ischemia/reperfusion led to a significant reduction in glycocalyx thickness in rat mesenteric venules [93]. Similarly, Bruegger et al. showed that in an ischemia-reperfusion study using isolated guinea-pig hearts, the impairment of endothelium-derived vasodilation was paralleled by disruption of the endothelial glycocalyx [122]. In humans, glycocalyx shedding was demonstrated in patients undergoing major vascular surgery with global or regional ischemia [124]. Interestingly, the importance of oxidative stress in this phenomenon was also suggested from studies showing that the effects of ischemia/reperfusion on the glycocalyx could be attenuated by a blockade of xanthine-oxidoreductase, which is an endogenous ROS producing enzyme bound to HS domains in the glycocalyx [121]. Taken together, these data support a role for endothelial glycocalyx in the pathophysiology of inflammatory response connected with ischemia/reperfusion-induced tissue damage.

### 7.4. Systemic Inflammation and Trauma

Systemic inflammatory response to microbial infection or to extensive tissue damage leads to serious pathological conditions such as sepsis and multiple organ failure [80]. These conditions are accompanied by the disruption of vessel functions mediated by various factors including the deregulated synthesis of NO and the massive release of proinflammatory cytokines, such as TNF-*α* [80, 125]. Leakage due to defects in endothelial barrier functions is one of the major clinical problems facing critically ill patients [80, 104]. It leads to the severe disturbance of microcirculation and consequently the failure of systemic circulation. In a complex study, Schmidt et al. demonstrated that endotoxemia in mice rapidly induced pulmonary microvascular glycocalyx degradation via TNF-*α*-dependent mechanisms involving the activation of endothelial heparanase [125]. This pulmonary endothelial glycocalyx degradation was connected with neutrophil adherence and inflammation and potentially with sepsis-associated respiratory failure [125]. In previous studies, experimentally induced endotoxemia in rats elicited plasma HA release and a reduction in endothelial surface thickness, indicative of glycocalyx degradation [126]. Similarly, experimentally induced endotoxemia in mice led to increased syndecan-1 plasma levels suggesting glycocalyx degradation [127]. In clinical studies, a directly determined decrease in glycocalyx thickness in critically ill patients was shown by Donati et al., who observed a more profound decrease in thickness in more severely septic patients [128]. Other authors showed that increased levels of glycocalyx components such as HS or syndecan-1 appear in the blood of septic shock and trauma patients [104, 125, 129–131], and that such increased levels were even significantly higher in nonsurvivors or positively correlated with increased mortality [104, 131]. Further, significantly increased levels of HS and syndecan-1 were observed in patients after major postabdominal surgery even without systemic inflammatory response syndrome [130]. Significant destruction of the glycocalyx soon after trauma induction is also suggested from data obtained by Ostrowski et al. with respect to severely injured patients [132].

## 8. Therapeutic Strategies to Ameliorate Glycocalyx Dysfunction 

As described above, under inflammatory conditions the integrity of the endothelial glycocalyx deteriorates to varying degrees particularly during generalized inflammatory responses of the body. To track the endogenous recovery of the glycocalyx* in vivo*, an interesting study was performed by Potter et al., who found that after acute enzymatic or cytokine-mediated degradation of the glycocalyx, 5 to 7 days were required for the glycocalyx to regain its original thickness. Thus the potentiation of this process should limit inflammatory processes in vessels. Positive effects of therapeutic strategies which would prevent shedding of glycocalyx components, prevent degradation of glycocalyx structure, and potentiate recovery of glycocalyx structure can be suggested for range of pathological conditions connected with inflammatory processes in the vessels mentioned above. Therapeutic strategies can be viewed from several perspectives. Principally, it is possible to differentiate between therapeutic strategies directly aimed at preserving, supporting, or reconstituting the glycocalyx structure and strategies with an indirect mechanism of action down regulating inflammatory processes which lead to glycocalyx structure damage.

### 8.1. Preservation of the Glycocalyx Structure by Exogenously Applied GAGs

The shedding of GAGs from the glycocalyx structure is observed both experimentally and in clinical trials. Thus, intravascular supplementation with sulfated polysaccharides to support glycocalyx structure or to reconstitute the endothelial glycocalyx seems intuitively advantageous. It can be speculated that increased levels of precursors of the glycoproteins in the glycocalyx might induce these glycoproteins to be regenerated.* In vivo*, heparin applied by intravenous injection was shown to interact rapidly and specifically with the endothelium [133]. Further, a combination of two glycocalyx abundant GAGs, HA and CS, administered by infusion was shown to partially regenerate the capillary glycocalyx damaged by hyaluronidase in hamsters [49]. Interestingly, treatment with either molecule separately had no effect [49]. Interestingly, also* in vitro*, Potter and Damiano observed a surface-bound GAG layer on cultured human umbilical vein endothelium only after supplementation of the culture medium with GAGs, HA and CS [53]. Constantinescu et al. demonstrated that intravascular supplementation with HS and heparin, but not dextran sulfate, attenuated ox-LDL-induced leukocyte-endothelial cell adhesion in mouse cremaster venules after degradation of the endothelial glycocalyx by local microinjection of heparitinase [77]. Interestingly, they showed that fluorescently labeled HS and heparin, but not dextran sulfate, became attached to the venule luminal surface after ox-LDL administration. Similarly, the intravenous application of heparin inhibited the negative effects of ischemia/reperfusion which were determined by intravital-microscopy in mouse cremasteric microvessels as the macromolecule exclusion and intracapillary distribution of red blood cells [121]. However, this effect was suggested to be connected with a decrease in ROS production, not directly with the incorporation of exogenous heparin into the glycocalyx structure.

Interestingly, sulodexide, a mixture of glycocalyx GAG precursors consisting of heparin sulphate (80%) and dermatan sulphate (20%), can be included among compounds suggested as a possible treatment of dysfunctional glycocalyx. [112]. It was shown by Gambaro et al. that long-term administration of this GAG preparation prevents renal morphological and functional alterations and appears to revert established diabetic renal lesions in experimental diabetic nephropathy induced in rats [134]. In clinical trials, Broekhuizen et al. demonstrated that sulodexide administration for 2 months increased glycocalyx thickness, which was found to be primarily altered in patients with type 2 diabetes compared to healthy controls, as was estimated in two different vascular beds using sidestream dark field imaging and combined fluorescein and indocyanine green angiography for sublingual and retinal vessels, respectively [112]. Although the reversal of glycocalyx abnormalities in diabetes was only partial, it would appear that this approach is promising. In another study, sulodexide therapy for a period of one year reduced albuminuria in diabetic patients [135]. The drug appeared active in both type 1 and type 2 diabetes and in both micro- and macroalbuminuric patients. No change in metabolic control and no systemic side effects were reported [135]. Finally, sulodexide showed significant cardioprotective effects after myocardial revascularization in rabbits [136]. However, in none of these studies did the authors determine effects on glycocalyx properties.

In contrast to the abovementioned studies, intravenous heparin challenge was associated with increased vascular leakage of dextrans and impaired arteriolar vasodilation in mouse [101]. Further, the intravenous application of heparin led to the immediate release of eight proteins shown to localize in the glycocalyx with HS binding properties in patients with nephropathy [137]. The authors speculate that this was due to a previously described release of glycocalyx-associated proteins owing to their competitive binding to exogenous heparin [137], which then negatively affected glycocalyx barrier properties and the mechanotransduction of shear stress to the endothelium [101]. Overall, these authors conclude that a heparin challenge can have adverse effects on vascular homeostasis and suggest that a perturbation in the glycocalyx might be involved in this phenomenon. However, the effect of heparin on intact glycocalyx could differ from its effect on altered glycocalyx, which is the case of the suggested positive effects of the sequestration of exogenous heparin on vessel function.

### 8.2. Preservation of the Glycocalyx Structure by Natural Plasma Protein Levels

The simplest suggested way to protect the glycocalyx is to maintain a sufficiently high concentration of plasma proteins [5, 6]. From a theoretical standpoint, Becker et al. predict that poorer mechanical stability of a protein-denuded glycocalyx, heightened susceptibility to attack by proteases, as well as secondary damage to the vessel wall incurred by the greater adherence of inflammatory cells, could be expected [5, 6]. The protective effect of the albumin supplementation on glycocalyx preservation in a model of transplantation-induced ischemia/reperfusion glycocalyx damage was presented by Jacob et al. [138]. Albumin supplementation significantly attenuated pronounced shedding of the glycocalyx as well as interstitial edema and the increased adhesion of leukocytes observed after a cold ischemia. Interestingly, experimental studies suggest that concentrations of albumin significantly lower than the physiological value may be sufficient to protect vascular integrity [139].

### 8.3. Inhibitors or Scavengers of Glycocalyx Damaging Noxes

In this part direct inhibition of factors involved in glycocalyx degradation is discussed.

### 8.4. Antioxidants and NO

Antioxidants have been found to protect tissues and organs from ischemia/reperfusion damage in innumerable studies. This action might significantly involve protection of the glycocalyx [48, 95, 121, 140]. However, no clinical studies that focused on preserving vascular permeability have yet used this approach convincingly. NO can play a specific role. It is an important signaling molecule for vascular cells. At the same time, low levels of NO are suggested to prevent oxidative cell damage. An experimental study by Bruegger et al. described the protective effect of NO, applied only during reperfusion, on maintaining the glycocalyx and the permeability barrier in the face of redox stress [122]. This latter action was exerted only if the glycocalyx was not destroyed enzymatically beforehand; thus, the direct radical scavenging action of NO was held responsible by the authors [122].

### 8.5. Inhibitors of Proteases

As discussed above, since proteases such as thrombin have been reported to support the cleavage of syndecan ectodomains, there could be an opening for the therapeutic use of protease inhibitors [36, 68, 141]. Among the tested inhibitors is doxycycline. In several studies, Mulivor and Lipowsky with coauthors demonstrated the inhibition of MMP activity by doxycycline that significantly reduced shedding of the glycocalyx and leukocyte adhesion to endothelial cells in response to inflammatory and ischemic stimuli [76, 86–88]. Another candidate is antithrombin III, a physiological inhibitor of numerous serine proteases naturally present in the glycocalyx, as described above. In several studies, Chappel at al. showed that antithrombin significantly protected the glycocalyx from TNF-*α* and ischemia/reperfusion-induced shedding in hearts [36, 67, 68, 142]. The glycocalyx protection was accompanied by reduced postischemic leukocyte adhesion in hearts, reduced vascular permeability, reduced coronary leak, and reduced interstitial edema [36, 67, 68, 142].

### 8.6. Approaches to Preserve Glycocalyx Structure with Indirect Mechanisms of Action (Modulating Processes Which Lead to Glycocalyx Structure Damage)

Other approaches that could be suggested to protect glycocalyx are based on modulation of processes that lead to glycocalyx damage. In the context of the above described detrimental effects of inflammatory processes on glycocalyx integrity, the reduction of inflammation processes by various types of drugs can be suggested as highly potential therapeutic approaches.

### 8.7. TNF-*α* Signaling Inhibition

TNF-*α* is one of the key mediators in the development of acute and chronic inflammation. Interestingly, a clinically used inhibitor of TNF-*α* signaling, Etanercept, an analog of the TNF-*α* receptor, significantly reduced the shedding of glycocalyx constituents, coagulation activation, and functional vessel function disturbances induced by experimental endotoxin application in humans [57].

### 8.8. Glucocorticoids

Glucocorticoids are routinely applied in the prevention of interstitial edema and swelling due to the substantial reduction in vessel permeability for macromolecules [5, 6, 36, 67, 68, 83, 142, 143]. The importance of the preservation of the glycocalyx is supported by results from an isolated heart model, in which preconditioning with hydrocortisone significantly reduced glycocalyx shedding following both ischemia/reperfusion and TNF-*α*-induced inflammation [36, 67, 83, 142]. Interestingly, clinical data show that hydrocortisone significantly reduced inflammatory response and the need for circulatory and ventilatory support in cardiac surgical patients, which was suggested to be associated with sustaining vascular barrier function, that is, with the prevention of the shedding of the glycocalyx [143].

Despite the positive effects of glucocorticoids, the exact mode of their action remains unclear [83]. Apart from the direct effect of hydrocortisone on endothelial cells, an inhibitory effect on immune effector cells has also been noted. Some authors underline the importance of the glucocorticoidal stabilization of mast cells, which should prevent degranulation and consequently abrogate proteolytic damage to the glycocalyx and the potentiation of inflammation [5, 6].

### 8.9. Volatile Anesthetic Sevoflurane and Isoflurane

Volatile anesthetics, such as sevoflurane and isoflurane, were suggested to pose anti-inflammatory effects and to ameliorate endothelial glycocalyx destruction induced by inflammatory response mediated by ischemia/reperfusion [144–146]. Sevoflurane was shown to pose complex anti-inflammatory effects on blood cells, leukocytes, and platelets and provided endothelial protection against ischemia/reperfusion injury* in vivo* [147–149]. A direct effect on endothelial cells was shown in an* in vitro* study demonstrating the immediate and delayed protective effects of isoflurane pretreatment on cytokine-induced injury in human endothelial cells [150]. The sevoflurane-mediated protection of endothelial glycocalyx destruction was demonstrated in ischemia/reperfusion-induced degradation experiments with guinea pig hearts, with both preconditioning and rapid postconditioning being successful [144–146]. These authors suggested that the mechanism involved the attenuation of lysosomal cathepsin B release [145].

### 8.10. Reduction of Hyperglycemia or Hypercholesterolemia

Since glycocalyx volume is suggested to be significantly reduced during hyperglycemia or hypercholesterolemia, as discussed above, the therapeutic normalization of blood glucose levels or blood lipid status could be proposed to prevent these alterations to glycocalyx structure. However, to our knowledge, there are currently no more reports that would provide experimental or clinical data demonstrating direct improvements in glycocalyx structure or function after treatment with glucose lowering drugs such as insulin.

The reduction of hypercholesterolemia by statins can be suggested. Interestingly, a partial recovery of the systemic glycocalyx volume compared to healthy controls was observed in patients with familial hypercholesterolemia after treatment with rosuvastatin [151]. However, the study does not clarify whether this was a direct effect of the statin on endothelial cells, or more related to the improved lipid status.

## 9. Conclusion 

A wide range of data, both experimental and clinical, demonstrates that the integrity of the endothelial glycocalyx deteriorates to varying degrees under inflammatory conditions, particularly during a generalized inflammatory response of the body. However, improvements in techniques that would allow valid studies of the microvascular glycocalyx to be made on isolated endothelial cells are required in order to definitively determine whether mechanotransduction is a function of the endothelial cytoskeleton or of the arterial glycocalyx [78].

Interestingly, data from experimental and clinical studies suggest the possibility of glycocalyx protection or preservation by various means. However, despite some positive results in several studies, skepticism exists about their clinical use in the near future. Since the pathological process occurs early in the inflammatory process, this strategy is theoretically problematic. However, based on current data from various models any therapeutic approach that would improve the glycocalyx structure and function would have a good potential to prevent the pathological processes connected with vascular inflammation.
